# Treatment of Intervertebral Disc Degeneration

**DOI:** 10.1111/os.13254

**Published:** 2022-04-29

**Authors:** Jingguo Xin, Yongjie Wang, Zhi Zheng, Shuo Wang, Shibo Na, Shaokun Zhang

**Affiliations:** ^1^ Department of Spinal Surgery The First Hospital of Jilin University Changchun China; ^2^ Jilin Engineering Research Center for Spine and Spinal Cord Injury Changchun China; ^3^ Department of Ophthalmology The Second Hospital of Jilin University Changchun China

**Keywords:** Conservative treatment, Intervertebral disc degeneration, Mesenchymal stem cells, Non‐coding rna, Surgical treatment

## Abstract

Intervertebral disc degeneration (IDD) causes a variety of signs and symptoms, such as low back pain (LBP), intervertebral disc herniation, and spinal stenosis, which contribute to high social and economic costs. IDD results from many factors, including genetic factors, aging, mechanical injury, malnutrition, and so on. The pathological changes of IDD are mainly composed of the senescence and apoptosis of nucleus pulposus cells (NPCs), the progressive degeneration of extracellular matrix (ECM), the fibrosis of annulus fibrosus (AF), and the inflammatory response. At present, IDD can be treated by conservative treatment and surgical treatment based on patients' symptoms. However, all of these can only release the pain but cannot reverse IDD and reconstruct the mechanical function of the spine. The latest research is moving towards the field of biotherapy. Mesenchymal stem cells (MSCs) are regard as the potential therapy of IDD because of their ability to self‐renew and differentiate into a variety of tissues. Moreover, the non‐coding RNAs (ncRNAs) are found to regulate many vital processes in IDD. There have been many successes in the in vitro and animal studies of using biotherapy to treat IDD, but how to transform the experimental data to real therapy which can apply to humans is still a challenge. This article mainly reviews the treatment strategies and research progress of IDD and indicates that there are many problems that need to be solved if the new biotherapy is to be applied to clinical treatment of IDD. This will provide reference and guidance for clinical treatment and research direction of IDD.

## Graphical abstract

The clinical treatment of IDD includes conservative therapy and surgical therapy. Currently, treatments based on MSCs and ncRNAs are regarded as potential targets. Some ncRNAs verified by clinical trials can be used in gene therapy, and drugs targeting those ncRNAs can also be designed to treat IDD.

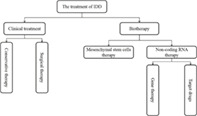

## Introduction

Intervertebral disc degeneration (IDD) is a pathological change defined as the aging process and damage of intervertebral disc (IVD) caused by a series of complex molecular mechanisms that finally leads to serious clinical symptoms. Low back pain (LBP) is one of the typical clinical symptoms of IDD. It is not only a common reason for patients to go to the hospital, but also one of the leading causes of disability.^1^, ^2^ Almost all people have transient attacks of LBP in their lives, and a small number of people will experience chronic LBP, which places a significant burden on the social economy, including not only the costs of treating patients (direct costs) but also the loss of social productivity (indirect costs).^3^, ^4^ IDD can be induced by a variety of factors, such as aging, heredity factors, mechanical loading, obesity, and even smoking.^5^, ^6^, ^7^, ^8^, ^9^, ^10^ The degeneration of IVD happens earlier than in other tissues of the body, as early in adolescence.^11^, ^12^ With the increase of age, the number of people affected by IVD increases sharply. About 10% of the 50‐year‐old population suffer from IDD, and in 70‐year‐old people, this number will increases to about 60%.^12^

**Fig. 1:**
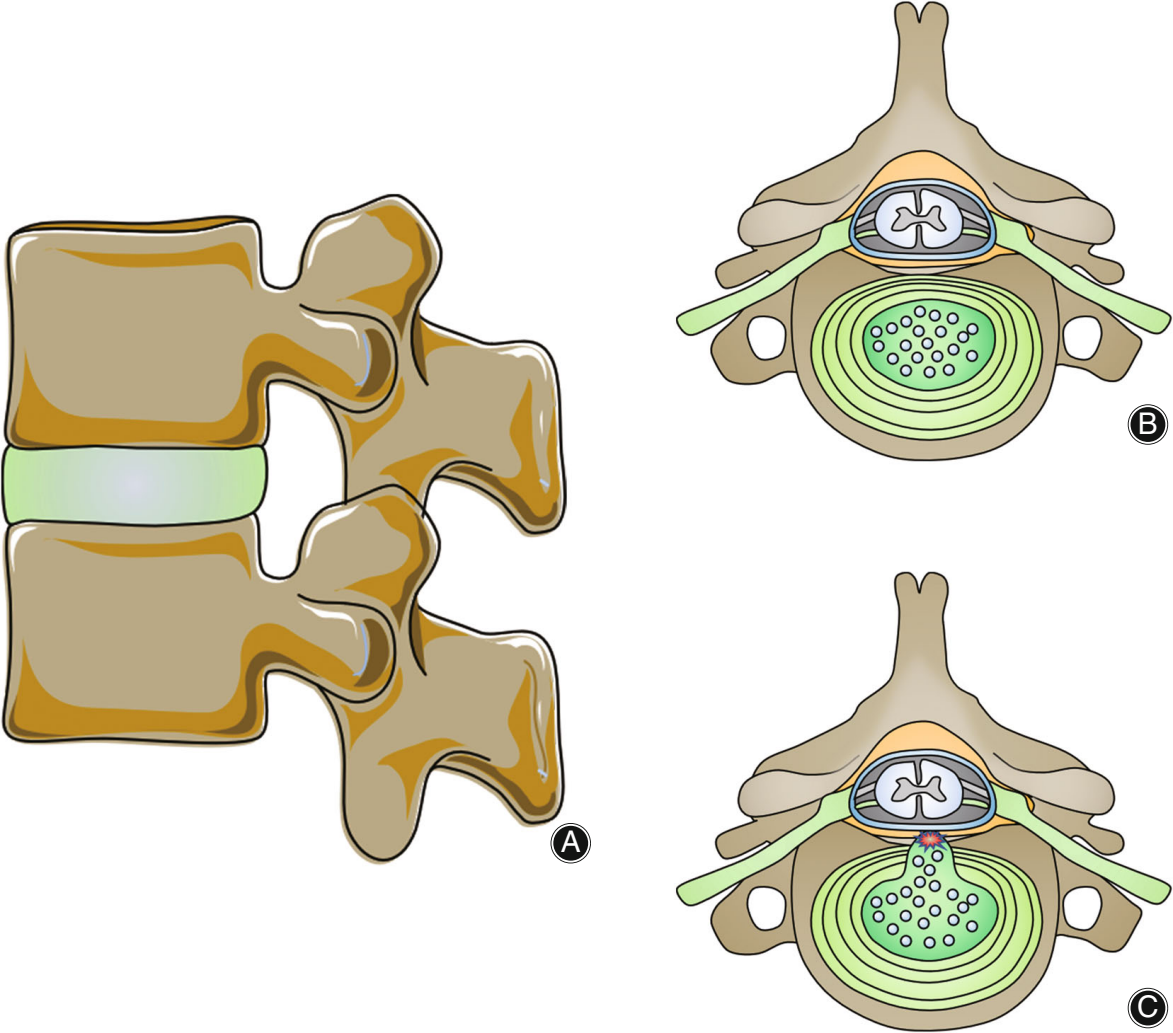
(A) The intervertebral discs firmly connect the upper and lower vertebrae and decompress, absorb shocks, and increase the range of spinal movement. (B) Three elements of intervertebral disc and adjacent structure. (C) Annulus fibrosus broke and nucleus pulposus herniate at the posterior part where near the spine canal.

IVDs are located between the upper and lower vertebrae, firmly connecting the centrums, and the IVDs can decompress, absorb shocks, and increase the range of spinal movement (Figure 1A). Each IVD consists of three elements: nucleus pulposus (NP), annulus fibrosus (AF), and cartilage endplate (CEP) (Figure 1B). In normal adults, there are few blood vessels in the IVD and most of the nutrient supply of IVD mainly depends on the infiltration from the CEP, which are the reasons why the IVD easily degenerates. The effect of IDD is mainly to change the movement and biomechanics of IVD, consequently affecting the mechanics of the spine. The intensity of normal IVD is primarily affected by the components of extracellular matrix (ECM). With age, the proteoglycan of ECM is lost and hydration ability decreases gradually, which leads to progressive dehydration of IVD, especially nucleus pulposus (NP).^7^, ^13^ Although the function of each IVD is about the same, according to the spinal cord segment in which it is located, their structure will change to adapt to the different stresses. The thickness and intensity of different parts of the AF are also different, for example, the posterior part of the lumbar intervertebral disc is thinner, and there is no collagen fiber interweaving in the posterior part, so the intensity of the posterior part is lower than other parts, which explains why disc herniation often occurs at the back of the IVD^14^ (Figure 1C). At the cellular level, environmental factors can accelerate the death of the cell through apoptosis or necrosis; these cellular processes, in turn, may further promote the pathogenesis of IDD.^15^ For example, the apoptosis and necrosis of NPCs not only reduce the number of functional cells but also release the inflammatory factors, which will further worsen the microenvironment of the IVD, leading to further degeneration of the IVD. Degeneration accumulation will cause clinical symptoms, then the patient needs to go to the hospital. At present, the main clinical treatments for IDD include conservative treatment and surgical treatment, which can relieve symptoms and reduce pain but cannot reverse the IDD, so the scientific community is beginning to study new biotherapy to delay or reverse the IDD. Many experiments *in vitro* have proved that biotherapy can promote the proliferation of NPCs, inhibit the fibrosis of IVD, preserve the water content and reverse the process of IDD. This article reviews the standard therapy and new biotherapy of IDD and puts forward the direction and prospect in the future.

## Currently Treatment

### Conservative Treatment

Conservative treatment is mainly suitable for the treatment of patients with early IDD, its main purpose is to relieve a patient's LBP and improve quality of life, which cannot cure the IVD, so it is regarded as palliative treatment. With the aggravation of IDD, conservative treatment often cannot control the clinical symptoms, and further surgical treatment will be taken.

### Drug Therapy

Currently, the commonly used drugs for the treatment of LBP are non‐steroidal anti‐inflammatory drugs (NSAIDs), opioid painkillers, muscle relaxants, benzodiazepines, antidepressants, corticosteroids, antiepileptic drugs, and so on. NSAIDs are widely used to treat LBP patients, including non‐selective NSAIDs and selective COX‐2 NSAIDs. A published Cochrane review suggested that NSAIDs are more effective than placebo for treating LBP (small magnitude) and low risk of side effects (maybe underestimated because of small sample size).^16^ Opioids are mainly used in patients with acute attacks, severe pain and are difficult to relieve. Constipation and sedation are the most common adverse symptoms,^17^ but the dosage and duration of opioids are controversial because of their addiction and central dependence. Muscle relaxants can relieve muscle spasm around the spine and are effective for patients with LBP.^18^ As adjunctive therapy, it could be more effective, however, with a higher risk of central nervous system adverse effects.^18^ Benzodiazepines have been used as muscle relaxants to treat LBP and the most common side effects are drowsiness and dizziness.^18^ Anoher random controlled trial suggested that benzodiazepines should be considered standard of care for patients with sciatica associated with lumbar disc prolapse.^19^ Regarding antidepressants, two systematic reviews reported that they can relieve physical pain,^20^, ^21^ but a randomized clinical trial suggested there was no difference in improvement in pain intensity between intervention group (low‐dose amitriptyline) and control group (placebo) after 6 months of treatment.^22^ Epidural steroid injections are one of the most common pain relief injections. Steroids inhibit the production of inflammatory chemicals in the body's immune system, which may be a source of pain. Chou *et al*.^23^ suggested that using epidural corticosteroid injections to treat spinal stenosis could reduce pain immediately but had no long‐term benefit. Antiepileptic drugs are also considered a useful treatment for LBP. In one study,^24^ the researchers chose topiramate for 48 patients with LBP and the results indicated that topiramate is a relatively safe and effective agent in the treatment of LBP. Taken together, these drugs have their unique effects and complications. In clinical practice, the severity of pain, the duration of symptoms, the risk factors of complications, and the cost of treatment should be considered when weighing and selecting treatment drugs. The drug chosen for the patient should be the best choice to balance all factors.

### Non‐drug Therapy

Non‐drug therapy mainly includes bed rest, traction, stent fixation, exercise therapy, acupuncture, massage, electromagnetic or electrothermal therapy, psychotherapy, and so on.^25^, ^26^, ^27^ These methods are used in many disciplines and fields and are often combined with drug therapy or surgery. Guo *et al*.^28^ studied the difference between low‐tension traction mode and high‐tension traction mode in traction therapy by establishing a mechanical degeneration model of IVD. The results showed that compared with high‐tension traction mode, low‐tension traction provides a stable microenvironment for the repairment of IVD and protects IVD functional cells better. And they further found that low‐tension traction combined with extracorporeal shock wave therapy could reduce the expression of MMP3, MMP13, and ADAMTS‐4.^29^ It is well‐known that MMPs and ADAMTS can promote collagen degradation, which leads to IDD. Therefore, low‐tension traction combined with extracorporeal shock wave therapy can further restore the microenvironment of degenerative IVD, reduce the tension of NP and AF, and alleviate the degeneration of IVD. After IDD, the biomechanical function of IVD decreased, and the spine lacked support and stability. Physical exercise can improve the strength of paraspinal muscles, provide support for the spine, and contribute to the proliferation of IVD cells. Buyukturan *et al*.^30^ conducted a randomized controlled trial that revealed that exercise can relieve pain and enhance muscle endurance. Transcutaneous Electrical Nerve Stimulation can also relieve pain according to two trials^31^, ^32^; however, it does not show any advantages compared with the exercise therapy. Although the above treatments cannot reverse the degenerative changes of IVD, they can relieve pain caused by IDD by stimulating cells, promoting metabolite transport, and preventing adhesion and re‐injury. In clinical practice, these treatments have a place in the treatment of patients with early iodine deficiency because they are non‐invasive and low‐cost.

### Interventional Treatment

The internal mechanics of the IVD can be changed and the neuropathic pain can be treated by heating, radiofrequency or injection of various chemicals into the IVD. These methods include intra‐discal electrothermal therapy (IDET),^33^, ^34^ radiofrequency myeloplasty,^35^ chemical nucleolysis by intradiscal injection such as ozone,^36^, ^37^ percutaneous discectomy,^38^, ^39^ and so on. These techniques hope to relieve a patient's symptoms by reconstructing the structure and shape of the IVD. Generally, the heating probe, radiofrequency probe or cutting device is introduced into the pathological area, which usually needs to be operated under the guidance of CT or fluoroscopy. Heating and radiofrequency can reduce inflammation and cause tissue contraction to reduce compression. In general, the common goal of these techniques is to reduce the pressure in the spinal canal, thereby liberating nerve root compression and reducing the clinical symptoms of patients. With the development of endoscope technology in spine surgery, these techniques have changed from non‐visual indirect reduction to visually direct operation. The possible mechanism of IVD injection of ozone in the treatment of IDD is that ozone can reduce the herniated NP and reduce the inflammatory reaction, thus reducing the pain of patients. Studies by Elawamy *et al*.^40^ and Ercalik *et al*.^41^ have shown that IVD injection of ozone can effectively control disc herniation and relieve the pain caused by it. After these conservative treatments, if a patient's symptoms cannot be controlled or are aggravated, further surgical treatments will be needed.

## Surgical Treatment

### Intervertebral Disc Fusion

Intervertebral disc fusion has always been regarded as the standard for surgical treatment of symptomatic IDD.^42^ The choice of the surgical approach includes anterior approach, posterior approach, posterolateral approach, and so on.^43^ Minimally invasive surgery and open surgery are both available, and the surgical technique is quite mature, which is suitable for the vast majority of patients with IDD. Some studies have found that in intervertebral disc fusion surgery, compared with open surgery, the clinical effect of minimally invasive surgery is similar to open surgery and has great advantages in reducing muscle edema and surgical bleeding and contributing to postoperative functional recovery.^44^, ^45^ Minimally invasive intervertebral disc fusion surgery uses a smaller skin incision and reduces paraspinal muscle peeling and soft tissue injury. Therefore, intraoperative blood loss and postoperative pain will be less, postoperative functional recovery will be faster, and hospital stay will be shorter.^44^ But, the minimally invasive approach takes longer operation time and requires more proficient operators than the open approach.

Intervertebral disc fusion surgery usually involves the following steps. First, the surgeon separates the tissue and removes the damaged IVD, cleans up the IVD space, then places the prepared cage into the intervertebral space, which provides additional support for the spine after the operation. Then pedicle screws are used to fix the upper and lower vertebrae. After completing this critical step, the operator uses X‐rays to observe and correct the angle of screw placement and check the position of the cage.^46^ If the operation is not effective or the movement pattern of the adjacent segments is changed, it may lead to further degeneration of additional IVD and motor segments. Once this happens, a second operation may be needed.

In terms of pain relief and functional improvement, the effect of intervertebral disc fusion is effective. Fritzell *et al*.^47^ conducted a clinical randomized controlled study with visual analog scale (VAS) score and Oswestry (OS) questionnaire as criteria for follow‐up. The results revealed that lumbar fusion surgery can effectively reduce pain and disability compared with non‐operative treatment. But the study is considered unreasonable because it compares routine care and fusion surgery rather than more comprehensive conservative treatments such as exercise therapy. After that, Brox *et al*.^48^ also designed a randomized controlled study involving 64 patients, which suggested that fusion surgery relieved pain and improved motor function effectively, but there was no significant statistical difference between conservative treatment and fusion surgery except OS index. The sample size of this study is small and the follow‐up time is only 1 year, so larger samples and longer studies are required to improve the recommendation evidence of intervertebral disc fusion.

Although intervertebral disc fusion can relieve discogenic pain caused by IDD and improve patients' disability, it can cause severe problems in the long run because it eliminates the movement of the adjacent vertebral body. When two vertebrae are fused, it seriously limits the damping effect of the IVD during motion, increasing the load and stress of the surrounding tissue and IVD, which will lead to the degeneration of other IVD in adjacent segments.^49^ Therefore, in the past few years there has been a growing interest in total disc replacement because it can maintain the movement of the centrum.

### Total Disc Replacement

Total disc replacement (TDA) was used by Fernström firstly in the 1960s.^50^ He implanted a stainless steel ball into 191 lumbar IVD and 13 cervical IVD of 125 patients, and the clinical effect was similar to that of intervertebral disc fusion. However, the sinking and squeezing of the ball caused severe complications. With the development of biomaterials and the advent of artificial IVD, disc replacement is successful. At present, a large number of studies have produced evidence about the safety and effectiveness of TDA and proved that it is not inferior to the clinical efficacy of intervertebral disc fusion and provides the mobility of lumbar segments that intervertebral disc fusion surgery cannot provide. Skold *et al*.^51^ compared TDA with intervertebral disc fusion. Although both operations had satisfactory results for pain relief, TDA showed a better trend at 5‐year follow‐up. The randomized controlled trial of Furunes *et al*.^52^ demonstrated that there was no significant difference in the increase in the rate of adjacent segment degeneration after TDA compared with the non‐operative group, indicating that TDA does not increase the risk of adjacent segment degeneration. Berg *et al*.^53^ used distortion‐compensated Roentgen analysis to evaluate the difference between TDA and intervertebral disc fusion. Clinically, the surgical results of the TDA group were better, but there was no conclusion to explain the difference in terms of activity.

Qualified intervertebral disc implants must meet these requirements^54^: (i) maintain or restore the IVD function; (ii) high mobility, low friction, and high wear‐resistance; (iii) high stability when long‐term fixation; (iv) the ability to perform postoperative imaging. Moreover, lumbar disc implants are different from the cervical, not only because the load magnitude of lumbar is greater than cervical but also because of the moving patterns difference.^54^

Compared with intervertebral disc fusion, there seems to be no significant difference in the incidence of surgical complications of TDA. One study^55^ indicated that there were more surgical approach‐related complications in the TDA group than in the LIF group, while another study^56^ showed that there was no significant difference between the two groups. Disc replacement‐related complications included facet dislocation, pedicle fracture, device dislocation, and vertebral split fracture.^57^, ^58^, ^59^, ^60^ Once complications occur, patients may experience postsurgical interventions. There are four types of interventions: revision, device removal, supplemental fixation, and reoperation.^61^ The risk of these interventions is time‐specific regardless if a patient is healthy or not.^61^

In the field of surgery, there are always contradictory data in the comparison of intervertebral disc replacement and fusion. Each operation is supported by corresponding evidence and it is also affected by the experience and ability of surgeons. In the actual diagnosis and treatment, in the case of ineffective conservative treatment, we can choose any kind of operation as long as it can reduce the pain of patients. Achieving good patient satisfaction is the most important.

## Biotherapy

Recent studies have explored how to use mesenchymal stem cells and gene therapy to prevent, slow down, or even reverse IDD, and may provide a new direction for the treatment of IDD. A brief overview of this section will be given below.

### Mesenchymal Stem Cell Therapy

Mesenchymal stem cells (MSCs) are pluripotent adult stem cells with the ability to self‐renew and differentiate into a variety of tissues, including bone marrow mesenchymal stem cells (BM‐MSCs), cartilage mesenchymal stem cells, muscle mesenchymal stem cells, and adipose mesenchymal stem cells.^62^, ^63^, ^64^ In the past few decades, the scientific community has devoted a lot of energy to the study of MCSs in order to apply them to the field of IDD, and some progress has been made.

### Molecular Mechanisms of Mesenchymal Stem Cells in IDD

In 2003, Sakai *et al*.^65^ found that BM‐MSCs can slow down rabbit IDD, which laid a theoretical foundation for the follow‐up study of BM‐MSCs in IDD. One of the applications of MSCs in the treatment of intervertebral disc degeneration is implanting cells directly into the damaged intervertebral disc. However, the degenerative intervertebral disc is characterized by hypoxia,^66^, ^67^ low glucose,^67^ increased acidity,^66^, ^67^ high osmotic pressure,^68^ mechanical load,^69^, ^70^ and increased inflammatory factors,^71^, ^72^ which has a great effect on the survival of MSCs in the intervertebral disc.^73^ At the same time, it is also a significant challenge for MSCs in the treatment of intervertebral disc degeneration. Therefore, it is thought that embedding cells with some kind of scaffold and implanting intervertebral disc can promote the inoculation, proliferation, and differentiation of MSCs. In one study^74^ rabbit BM‐MSCs were loaded with novel nanofibre sponge microspheres to regenerate NP. The results showed that the complex of this material and BM‐MSCs could promote BM‐MSCs to differentiate into NP phenotype, produce ECM, maintain IVD height, and prevent IVD calcification.

The molecular mechanism of MSCs in the treatment of IDD is very complex. Xu *et al*.^75^ found that overexpression of BMP7 can promote BM‐MSCs to differentiate into NPCs, promote the differentiation and proliferation of NPCs in rabbit IVD, and restore the homeostasis of ECM. At the same time, these effects can be inhibited by Smad1 silencing. Yang *et al*.^76^ found that BM‐MSCs can secrete an anti‐inflammatory protein, TSG‐6, which can inhibit TLR2/NF‐κB pathway to reduce the production of inflammatory factors IL‐6 and TNF‐α, and then delay the inflammatory response in IDD. Growth differentiation factor 6 (GDF6) was found that could promote MSCs differentiation towards NPC type and increase ECM components expression.^77^

### Mesenchymal Stem Cell‐related Research in Treating IDD

As mentioned earlier, MSCs is a very potential site for the treatment of IDD. At present, there have been corresponding clinical trials and case reports on the use of MSCs in the treatment of patients with IDD. Kumar *et al*.^78^ conducted a 12‐month phase 1 clinical trial in which autologous adipose‐derived mesenchymal stem cells were obtained from 10 patients and injected into the IVD tissue of patients in conjunction with a hyaluronic acid derivative Tissuefill. Finally, the VAS pain score and ODI score of six patients improved significantly, of which three patients had intervertebral disc rehydration. Pettine *et al*.^79^ recruited 26 patients to receive autologous BM‐MSCs injections. Pain symptoms were alleviated in all subjects, and eight patients improved a Pfirrmann grade. They also suggested that the efficacy of the treatment was related to the concentration of the cells injected. Elabd *et al*.^80^ recruited five patients to receive autologous BM‐MSCs. All patients were followed up for more than 4 years, and all patients showed overall improvement in symptoms, which indicated that BM‐MSCs therapy was safe in the long term.

Although encouraging achievements have been made in the application of MSCs in the treatment of IDD, there are still some problems. First of all, we still need to further study the molecular mechanism of MSCs in the treatment of intervertebral disc degeneration, including their targets and signal pathways, some biomolecules that can promote the efficacy of MSCs and establish a complete molecular mechanism structure. Finally, a large number of high‐quality clinical studies are still needed to test the feasibility and safety of MSCs therapy.

## Non‐coding RNA Therapy

### Non‐coding RNAs and its roles in IDD

Non‐coding RNAs (NcRNAs), which include microRNAs (miRNAs), circRNAs, and long non‐coding RNAs (lncRNAs), cannot be transcribed and translated into proteins, but play an important role in molecular regulation. Among them, miRNAs generally directly target a protein or signal pathway to play its regulatory function. circRNAs and lncRNAs generally act as miRNAs sponges and regulate the expression of miRNAs. At present, it has been confirmed that ncRNAs play a vital role in the treatment of tumour^81^ and cardiovascular disease,^82^ and more and more people study its role in IDD. In recent years, more and more data have shown that there are significant differences in the expression of some ncRNAs between the IVD tissues of patients with IDD and that of normal persons, indicating that ncRNAs are involved in regulating the formation of IDD, some promote the occurrence of IDD, and some inhibit the occurrence of IDD.

### Non‐coding RNAs and its targets in IDD

There are a variety of molecular mechanisms and biological effects of ncRNAs in regulating IDD (Table S1).^83^, ^84^, ^85^, ^86^, ^87^, ^88^, ^89^, ^90^, ^91^, ^92^, ^93^, ^94^, ^95^, ^96^, ^97^, ^98^, ^99^, ^100^, ^101^, ^102^, ^103^, ^104^, ^105^, ^106^, ^107^, ^108^, ^109^, ^110^, ^111^, ^112^, ^113^, ^114^, ^115^, ^116^, ^117^, ^118^, ^119^, ^120^, ^121^, ^122^, ^123^, ^124^, ^125^, ^126^, ^127^, ^128^, ^129^, ^130^, ^131^, ^132^, ^133^, ^134^, ^135^, ^136^, ^137^, ^138^, ^139^, ^140^, ^141^, ^142^, ^143^, ^144^, ^145^, ^146^, ^147^, ^148^, ^149^, ^150^, ^151^, ^152^, ^153^, ^154^, ^155^, ^156^, ^157^, ^158^, ^159^, ^160^, ^161^, ^162^, ^163^, ^164^, ^165^, ^166^, ^167^, ^168^, ^169^, ^170^, ^171^ NcRNAs plays a vital role in cell proliferation, cell apoptosis, cell autophagy, ECM degeneration or degradation, inflammation, and so on. Bcl‐2 is a protein related to the regulation of cell apoptosis, and the apoptosis of NPCs is an important link in IDD, so Bcl‐2 is also an important target in IDD. miRNA‐143,^83^ miRNA‐155,^84^ and miRNA‐222^85^ have been reported to regulate the apoptosis of NPCs by targeting Bcl‐2. lncRNA‐GAS5 acts as a miRNA‐155 sponge to regulate its expression.^84^ On the other hand, the degeneration and degradation of ECM is also an important link in IDD. The MMP family is a zinc‐dependent metallopeptidase family that participates in the degradation of ECM components. Therefore, the MMP family is also a research hotspot in the field of IDD. It is reported that miRNA‐202‐3p targeting MMP1; miRNA‐17‐3p, miRNA‐93 targeting MMP2^87^, ^88^ and MMP3 is the target of miRNA‐31‐5p,^89^ which are involved in the regulation of ECM degradation of IVD. Moreover, miRNA‐133a target MMP9 inducing the loss of type Ⅱ collagen which is the important component of ECM.^90^ Other MMPs are also reported that relating to the ECM degradation and causing IDD such as MMP13, MMP14 and MMP16.^91^, ^92^, ^93^, ^94^ Interestingly, some miRNAs have been reported many times to target different pathways or related proteins involved in the regulation of different aspects of IDD. miRNA‐155 can target TCF7L2,^95^ C/EBPβ,^96^ ERK1/2,^97^ and MMP16^94^ involved in regulating the degradation of ECM and the expression of inflammatory factors in the inflammatory response.

As mentioned earlier, ncRNA is also a potential unit for the treatment of IDD, but how to transform the relationship between ncRNA and IDD into practical treatment is an urgent problem. It is also not known whether the use of ncRNA in the treatment of human IDD can achieve the same effect as at the cellular level and in animal experiments.

## Conclusion and Future Perspective

IDD is a common clinical degenerative disease, which can easily lead to low back pain, disc herniation, and other diseases, seriously affecting the quality of life of patients and bringing great economic burden to the society. At present, there are standard treatments for intervertebral disc degeneration in clinic, but these treatments encounter a “bottleneck,” that is, they can not reverse the occurrence of IDD, but can only relieve the pain of patients. Treatments based on MSCs and ncRNA are potential targets for the treatment of IDD and are of great research value. The molecular mechanism of MSCs in the treatment of IDD needs to be further explored, and a large number of clinical trials need to be designed to verify its feasibility and safety. Quite a number of ncRNA experiments have been carried out, but the role of some ncRNA in IDD is still controversial and needs to be verified by further experiments. Verified by reliable clinical trials, these reported data can be directly used in gene therapy for IDD, and drugs targeting ncRNA can also be designed to provide new ideas for the treatment of IDD.

## Conflict of Interests

The authors have declared that there are no competing interests.

## Supporting information


**Table S1** Recent 5‐year research progress related to ncRNA and IDD
