# Supplementary material for: Treatment of Intervertebral Disc Degeneration
Source: Orthop Surg. 2022 Apr 29;14(7):1271–80. doi: 10.1111/os.13254 (PMC9251272; doi:10.1111/os.13254)
Supplement: Supplementary file 1 — Table S1 Recent 5‐year research progress related to ncRNA and IDD [file OS-14-1271-s001.docx]

Supplement

Table S1. Recent five years research progress related ncRNA and IDD.

| ncRNA | Targets/Signal pathway | Biological functions | Research materials | Protect against/  Promote IDD | References |
| --- | --- | --- | --- | --- | --- |
| circRNA-104670 | miRNA-17-3p  MMP2 | promotes NPCs apoptosis  inhibits NPCs proliferation  inhabits collagen II expression | Human NPCs | Promote | Song et al.^87^ |
| circRNA-4099 | miRNA-616-5p  SOX9 | promotes collagen II expression  promotes aggrecan expression | Human NPCs | Protect | Wang et al.^98^ |
| circRNA-CIDN | miRNA-34a-5p  SIRT1 | inhabits NPCs apoptosis  inhabits ECM degradation | Human NPCs | Protect | Xiang et al.^99^ |
| circRNA-ERCC2 | miRNA-182-5p  SIRT1 | inhibits NPCs apoptosis  inhibits AF fibrosis  inhibits ECM degradation | Human NPCs  IDD rat model | Protect | Xie et al.^100^ |
| circRNA-GRB10 | miRNA-328-5p  ERBB2 | inhibits NPCs apoptosis | Human NPCs | Protect | Guo et al.^101^ |
| circRNA-SEMA4B | miRNA-431  SFRP1  GSK-3β | promotes collagen II expression  promotes aggrecan expression  promotes NPCs proliferation  inhabits NPCs senescence | Human NPCs | Protect | Wang et al.^102^ |
| circRNA-VMA21 | miRNA-200c  XIAP | promotes collagen II expression  promotes aggrecan expression  inhabits NPCs apoptosis | Human NPCs  IDD rat models | Protect | Cheng et al.^103^ |
| lncRNA-FAF1 | Erk signaling pathway | promotes NPCs proliferation | Human NPCs | Promote | Mi et al.^104^ |
| lncRNA-FAM83H-AS1 | Notch1 | inhabits collagen II expression  inhabits aggrecan expression  promotes NPCs proliferation | Human NPCs | Promote | Wei et al.^105^ |
| lncRNA-GAS5 | miRNA-155  Bcl-2  caspase‑3 | promotes NPCs apoptosis | Human NPCs | Promote | Wang et al.^84^ |
| lncRNA-H19 | miRNA-22  LEF1 | promotes collagen I expression  inhabits NPCs proliferation | Human NPCs | Promote | Wang et al.^106^ |
| lncRNA-HCG18 | miRNA-146a-5p  TRAF6 | promotes NPCs apoptosis  inhabits NPCs growth | Human NPCs | Promote | Xi et al.^107^ |
| lncRNA-HOTAIR^†^ | Wnt/β-catenin signaling pathway | promotes NPCs senescence  promotes NPCs apoptosis  promotes ECM degradation | Human NPCs  IDD rat models | Promote | Zhan et al.^108^ |
| lncRNA-HOTAIR^†^ | miRNA-34a-5p  Notch1 | inhabits NPCs apoptosis | Human NPCs | Protect | Shao et al.^109^ |
| lncRNA-linc00641 | miRNA-153-3p  ATG5 | promotes collagen II expression  inhibits NPCs autophagy | Human NPCs | Protect | Wang et al.^110^ |
| lncRNA-linc00958 | miRNA-203 | inhabits collagen II expression  inhabits aggrecan expression  promotes NPCs proliferation | Human NPCs | Promote | Zhao et al.^111^ |
| lncRNA-linc00969 | miRNA-335-3p  TXNIP | promotes NPCs apoptosis | Human NPCs | Promote | Yu et al.^112^ |
| lncRNA-lincADAMTS5 | RREB1 | inhabits ECM degradation | Human NPCs | Protect | Wang et al.^113^ |
| lncRNA-lincRNA-SLC20A1 | miRNA-31-5p  MMP3 | promotes ECM degradation  inhabits collagen II expression  inhabits aggrecan expression | Human NPCs | Promote | Yang et al.^89^ |
| lncRNA-MALAT1^†^ |  | promotes CEP cells apoptosis | Rat CEP cells | Promote | Jiang et al.^114^ |
| lncRNA-MALAT1^†^ |  | promotes NPCs proliferation  inhabits NPCs apoptosis  inhabits IL-1 expression  inhabits IL-6 expression | Human NPCs | Protect | Zhang et al.^115^ |
| lncRNA-NEAT1 | MAPK signaling pathway | promotes ECM degradation | Human NPCs | Promote | Ruan et al.^116^ |
| lncRNA-PART1 | miRNA-93  MMP2 | promotes NPCs apoptosis  inhabits collagen II expression  inhabits aggrecan expression | Human NPCs | Promote | Gao et al.^88^ |
| lncRNA-RP11-296A18.3 | miRNA-138  HIF1A | inhabits NPCs proliferation  promotes collagen I expression | Human NPCs | Promote | Wang et al.^117^ |
| lncRNA-SNHG1 | miRNA-326  CCND1 | promotes NPCs proliferation | Human NPCs | Promote | Tan et al.^118^ |
| lncRNA-TRPC7-AS1 | miRNA-4769-5p  HPN 3′UTR | promotes NPCs senescence  inhabits NPCs viability  inhabits ECM synthesis | Human NPCs | Promote | Wang et al.^119^ |
| lncRNA-TUG1 | Wnt/β-catenin signaling pathway | promotes NPCs apoptosis  promotes NPCs senescence  inhabits NPCs proliferation | Human NPCs | Promote | Chen et al.^120^ |
| miRNA-106a-5p | ATG7 | promotes AFCs apoptosis | Human AFCs | Promote | Hai et al.^121^ |
| miRNA-107 | HMGB1 | inhabits inflammatory response | Human NPCs | Protect | Niu et al.^122^ |
| miRNA-125a | TP53INP1 | inhabits NPCs apoptosis | Human NPCs | Protect | Ma et al.^123^ |
| miRNA-127-5p | MMP13 | inhabits collagen II catabolism | Human NPCs | Protect | Hua et al.^91^ |
| miRNA-129-5p | BMP2 | promotes NPCs viability  inhabits NPCs apoptosis | Human NPCs | Protect | Yang et al.^124^ |
| miRNA-132 | GDF5 | promotes ECM degradation | Human NPCs  IDD rat model | Promote | Liu et al.^125^ |
| miRNA-133a | MMP9 | promotes collagen II expression | Human NPCs | Protect | Xu et al.^90^ |
| miRNA-138-5p | SIRT1 | promotes NPCs apoptosis | Human NPCs | Promote | Wang et al.^126^ |
| miRNA-140-5p | TLR4 | inhibits IVD inflammation | Human NPCs | Protect | Zhang et al.^127^ |
| miRNA-141 | SIRT1 | inhabits NPCs proliferation  promotes NPCs apoptosis | Human NPCs  IDD rat model | Promote | Ji et al.^128^ |
| miRNA-143 | Bcl-2 | promotes NPCs apoptosis | Human NPCs | Promote | Zhao et al.^83^ |
| miRNA-143-5p | eEF2 | inhabits collagen II expression  inhabits aggrecan expression  inhibits NPCs proliferation  and differentiation  promotes NPCs apoptosis  and senescence | IDD rat model | Promote | Yang et al.^129^ |
| miRNA-145 | ADAM17 | inhabits NPCs apoptosis  promotes ECM synthesis | Human NPCs  Rat NPCs | Protect | Zhou et al.^130^ |
| miRNA-146a | TRAF6 | inhabits TNF-α, IL-1β, IL-6 expression | Human NPCs | Protect | Lv et al.^131^ |
| miRNA-149 | MyD88 | promotes collagen II expression  promotes aggrecan expression  inhabits inflammatory cytokines expressions  inhabits NPCs apoptosis | Rat NPCs | Protect | Qin et al.^132^ |
| miRNA-150 | P2X7 | inhibits ECM catabolism  inhibits inflammatory responses  inhibits NPCs apoptosis | Human NPCs  IDD rat model | Protect | Zhang et al.^133^ |
| miRNA-155 | TCF7L2 | inhibits ECM degradation | Human NPCs  Rat NPCs | Protect | Sun et al.^95^ |
| miRNA-155 | C/EBPβ | inhabits catabolic genes expression | Rat NPCs | Protect | Zhou et al.^96^ |
| miRNA-155 | ERK1/2 | promotes collagen II expression  promotes glycosaminoglycan expression | Human NPCs | Protect | Ye et al.^97^ |
| miRNA-155 | MMP16 | promotes collagen II expression  promotes aggrecan expression | Human NPCs | Protect | Zhang et al.^94^ |
| miRNA-15a | MAP3K9 | inhabits NPCs proliferation  promotes NPCs apoptosis | Human NPCs | Promote | Cai et al.^134^ |
| miRNA-15b | SMAD3 | promotes ECM degradation | Human NPCs | Promote | Kang et al.^135^ |
| miRNA-184 | GAS1 | promotes NPCs proliferation | Human NPCs | Promote | Li et al.^136^ |
| miRNA-193a-3p | MMP14 | promotes collagen II expression | Human NPCs  IDD rat model | Protect | Ji et al.^93^ |
| miRNA-194 | TRAF6 | inhabits inflammatory cytokines expression  promotes collagen II expression  promotes aggrecan expression | Rat NPCs | Protect | Kong et al.^137^ |
| miRNA-194-5p | CUL4A  CUL4B | inhabits IDD by downregulating CUL4A and CUL4B expression | Human osteoblast cell line Human AFCs  Human NPCs  Venous blood samples | Protect | Chen et al.^138^ |
| miRNA-202-3p | MMP1 | promotes the fluorescence intensity of type II collagen | Human NPCs | Protect | Shi et al.^86^ |
| miRNA-20a | ANKH | promotes CEP chondrocytes mineralization | Human CEP cells | Promote | Liu et al.^139^ |
| miRNA-21^†^ | PTEN | inhabits collagen II expression  inhabits aggrecan expression | Human NPCs | Promote | Wang et al.^140^ |
| miRNA-21^†^ | PTEN | inhibits NPC apoptosis | Human NPCs  Human MSCs  IDD rat model | Protect | Cheng et al.^141^ |
| miRNA-21^†^ | PDCD4 | promotes NPCs proliferation | Human NPCs | Promote | Chen et al.^142^ |
| miRNA-210 | ATG7 | inhabits collagen II expression  inhabits aggrecan expression  inhibits degenerated NPCs autophagy | Human NPCs | Promote | Wang et al.^143^ |
| miRNA-221^†^ | TRPS1 | inhabits collagen II expression  inhabits aggrecan expression | Human IVD cells | Promote | Penolazzi et al.^144^ |
| miRNA-221^†^ | FOXO3 | inhabits collagen II expression  inhabits aggrecan expression | Human IVD cells | Promote | Penolazzi et al.^145^ |
| miRNA-221^†^ | ERα | inhabits collagen II expression  inhabits aggrecan expression  promotes NPCs apoptosis | Human CEP cells | Promote | Sheng et al.^146^ |
| miRNA-221^†^ | Smads（1,5,8） | promotes AFCs proliferation  diminishes the osteogenic potential of degenerated AFCs | Human AFCs | Protect | Yeh et al.^147^ |
| miRNA-222 | Bcl-2 | inhibits NPCs proliferation  promotes NPCs apoptosis | Human NPCs | Promote | Wang et al.^85^ |
| miRNA-222 | TIMP3 | promotes TNF-α, IL-1β,  IL-6 expression  inhabits collagen II expression  inhabits aggrecan expression  promotes NPCs apoptosis | Human NPCs | Promote | Zhang et al.^148^ |
| miRNA-222-3p | CDKN1B | promotes NPCs apoptosis  inhabits NPCs proliferation  inhabits collagen II expression  inhabits aggrecan expression | Human NPCs | Promote | Liu et al.^149^ |
| miRNA-223 | Irak1 | inhibits inflammatory responses | Rat NPCs | Protect | Wang et al.^150^ |
| miRNA-2355-5p | ERRFI1 | promotes NPCs growth  promotes pro-inflammatory cytokine production | Human NPCs | Promote | Guo et al.^151^ |
| miRNA-23c | GSK3B | inhabits NPCs proliferation  inhabits collagen II expression  inhabits aggrecan expression | Human NPCs | Promote | Yang et al.^152^ |
| miRNA-24-3p | IGFBP5 | promotes NPCs apoptosis | Human NPCs | Promote | Chen et al.^153^ |
| miRNA-25-3p | Bim | promotes NPCs proliferation  inhabits NPCs apoptosis | Rat NPCs | Protect | Zhao et al.^154^ |
| miRNA-26a-5p | SMAD1 | promotes VEGF‐A expression | Rat serum sample  Rat NPCs  Rat AFCs | Promote | Fan et al.^155^ |
| miRNA-27b | MMP13 | promotes collagen II expression | Human NPCs | Protect | Li et al.^92^ |
| miRNA-30d | SOX9 | inhabits NPCs viability  promotes NPCs apoptosis  inhabits collagen II expression  inhabits aggrecan expression | Human NPCs | Promote | Lv et al.^156^ |
| miRNA-3150a-3p | ACAN | inhabits aggrecan expression | Human NPCs | Promote | Zhang et al.^157^ |
| miRNA-34a | GDF5 | inhabits collagen II expression  inhabits aggrecan expression | Human NPCs | Promote | Liu et al.^158^ |
| miRNA-365 | HDAC4 | promotes end plate chondrocyte proliferation  inhabits end plate chondrocyte degeneration | Human Chondrocytes | Protect | Zheng et al.^159^ |
| miRNA-455-5P | RUNX2 | promotes the stress resistance  of endplate chondrocytes | Human Chondrocytes | Protect | Xiao et al.^160^ |
| miRNA-483-3p | CTNNB1 | promotes NPCs proliferation  promotes collagen II expression  promotes aggrecan expression | Human NPCs | Protect | Yang et al.^152^ |
| miRNA-486-5p | FOXO1 | inhabits TNF-α, IL-1β,  IL-6 expressions  promotes collagen II expression  promotes aggrecan expression | Human NPCs | Protect | Chai et al.^161^ |
| miRNA-499a-5p | SOX4 | inhabits NPCs apoptosis  promotes collagen II expression  promotes aggrecan expression | Human NPCs | Protect | Sun et al.^162^ |
| miRNA-532 | Bcl-9 | promotes NPCs apoptosis | Human NPCs  Human blood sample | Promote | Sun et al.^163^ |
| miRNA-573 | Bax | promotes cell viability  inhibits NPCs apoptosis | Human NPCs | Protect | Wang et al.^164^ |
| miRNA-640 | LRP1 | promotes TNF-α, IL-1β production  promotes NPCs degeneration | Human NPCs  Human AFCs | Promote | Dong et al.^165^ |
| miRNA-660 | SAA1 | promotes NPCs apoptosis | Human NPCs | Promote | Zhang et al.^166^ |
| miRNA-665 | GDF5 | inhabits collagen II expression  inhabits aggrecan expression  promotes NPCs proliferation | Human NPCs | Promote | Tan et al.^167^ |
| miRNA-7 | GDF5 | promotes ECM degeneration | Human NPCs | Promote | Liu et al.^168^ |
| miRNA-96 | FRS2 | inhibits NPCs proliferation  promotes NPCs apoptosis | Human NPCs | Promote | Yang et al.^169^ |
| miRNA-96 | ARID2 | promotes degenerated NPCs proliferation | Human NPCs | Promote | Tao et al.^170^ |
| miRNA-98 | IL-6 | promotes collagen II expression  promotes NPCs proliferation  inhabits NPCs apoptosis | Human NPCs | Protect | Ji et al.^171^ |

Abbreviation：ACAN, aggrecan; ADAM, a disintegrin and metalloproteinase; ANKH, ankylosis protein homolog; ARID, AT‑rich interaction domain; ATG, autophagy-related gene; Bcl-2, B-cell lymphoma-2; Bim, Bcl-2 interacting mediator of cell death; BMP, bone morphogenetic protein; C/EBPβ, CCAAT/enhancer binding protein β; CDKN1B, Cyclin-dependent kinase inhibitor 1B; CUL, cullin; eEF2, eukaryotic elongation factor 2; ERRFI1, ErbB receptor feedback inhibitor 1; ERα, estrogen receptor alpha; FOXO1, forkhead box protein O1; FRS2, fibroblast growth factor receptor substrate 2; GAS1, Growth Arrest Specific Gene 1; GDF5, growth differentiation factor5; HDAC4, Histone deacetylase 4; HIF1A, hypoxia-inducible factor 1-alpha; HMGB1, high-mobility group box 1; HPN, Hepsin; IGFBP5, insulin-like growth factor-binding protein 5; IL-6, Interleukin-6; LEF1, lymphoid enhancing factor-1; LRP1, LDL receptor‐related protein‐1; MMP, matrix metalloproteinase; PDCD4, programmed cell death 4; PTEN, phosphatase and tensin homolog; RREB1, Ras-responsive element-binding protein 1; RUNX2, Runt‐related transcription factor 2; SAA1, Serum amyloid A1; SFRP1, secreted Frizzled-related protein 1; SIRT1, silent mating type information regulation 2 homolog 1; SMAD3, Mothers against decapentaplegic homolog 3; SOX, sex-determining region Y-box; TCF7L2, Transcription factor 7-like 2; TIMP3, tissue inhibitor of metalloproteinase 3; TLR4, Toll-like receptor 4; TP53INP1, Tumor protein 53 induced nuclear protein 1; TRAF6, tumor necrosis factor receptor-associated factor 6; TRPS1, trichorhinophalangeal syndrome 1; TXNIP thioredoxin-interacting protein; XIAP, X linked inhibitor-of-apoptosis protein.

† indicates that the effect of this kind of ncRNA on IDD is controversial or there are contradictory results.
